# Communicating antimicrobial resistance and stewardship in the national press: Lessons from sepsis awareness campaigns

**DOI:** 10.1016/j.jinf.2018.09.001

**Published:** 2019-02

**Authors:** L. Rush, C. Patterson, L. McDaid, S. Hilton

**Affiliations:** MRC/CSO Social and Public Health Sciences Unit, University of Glasgow, Scotland, United Kingdom

**Keywords:** Drug resistance, microbial, Sepsis, Mass media

## Abstract

• Antimicrobial resistance (AMR) and sepsis have been the subject of increasing media focus.
• Reporting of these issues in UK newspapers contains potentially conflicting messages about antibiotic use.
• Articles about sepsis frequently document its impact using personal narratives that rarely feature in articles about AMR.
• Few articles balance messages about early antibiotic treatment in sepsis with the need to reduce unnecessary prescribing.
•   Integrating media discourses about AMR and sepsis may help improve public understandings about the importance of antimicrobial stewardship.

## Introduction

Clear messages from the scientific community warn of the threat to human health from increasing ineffectiveness of antibiotics as a result of antimicrobial resistance (AMR). The World Health Organisation (WHO) has set out strategic goals to address AMR at a global level, and in the United Kingdom (UK), a report by the Review on AMR^1^ predicted ten million deaths from resistant infections annually by 2050.^1^^,^^2^ There have been repeated warnings about its potential impact on healthcare, with multiple important medical interventions likely to be jeopardised by a lack of effective antibiotics.^3^

Concurrently, there has been increasing focus on improving outcomes for patients with sepsis, a serious complication of untreated bacterial infection. Internationally, the Surviving Sepsis Campaign aims for better implementation of treatment guidelines, while in the UK the Sepsis Trust campaigns for improved awareness among the public, health professionals and policymakers.^4^^,^^5^ Early administration of antibiotics is central to improving outcomes, with survival reduced by 7.6% each hour that treatment is delayed.^6^ Two high-profile reports identified that delays in initiating appropriate management are widespread within the UK healthcare system.^7^^,^^8^ In 2016, National Institute for Health and Clinical Excellence (NICE)^2^ guidance urged health professionals to consider sepsis in any patient exhibiting signs of infection, in the same way they would consider myocardial infarction in patients with chest pain.^9^

Diagnosing sepsis is challenging, particularly in primary care without laboratory-based parameters to help stratify risk. Some diagnostic uncertainty is unavoidable, and NICE acknowledges that there is a lack of evidence regarding which patients can be sent home safely and which need intravenous or oral antibiotics, and that wrong prescribing decisions can have “catastrophic” consequences.^9^

Estimates of deaths from sepsis are subject to uncertainty due to limitations of current surveillance systems, as is the proportion of cases caused by drug-resistant infections.^10^ Reducing unnecessary antibiotic use through antimicrobial stewardship measures that include patient education, training for health professionals and regular audit and feedback of prescribing is essential to ensure that effective antibiotics remain available to treat sepsis in the long-term. Yet in the short-term, this aim could be interpreted as in conflict with professional guidance that recommends a low threshold for considering sepsis, particularly in the context of evidence that, for health professionals, preventing harm in individual patients overrides longer-term societal benefits of stewardship.^11^^,^^12^

Both AMR and sepsis have been subjects of substantial media focus within the UK, with extensive coverage of certain cases of sepsis, notably one-year-old William Mead who died in 2014 following complications associated with a streptococcal infection. A public enquiry led by NHS England^3^ concluded that pressure on general practitioners (GPs) to avoid unnecessary prescribing of antibiotics may have been a contributory factor in his death.^13^ Media framing of health issues can influence public understandings by assigning different degrees of salience to specific aspects, opinions and risks reported and has been shown to have the capacity to influence decision-making about healthcare, for example in relation to vaccine uptake.^14^^,^^15^ Newspaper coverage of AMR and sepsis may contain conflicting messages about when antibiotics are required that may be confusing for the public. If UK newspapers’ constructions of sepsis influence individuals’ perceptions of risk, they may drive demand for unnecessary antimicrobial treatment, with implications for antimicrobial stewardship. Previous analyses of media representations of AMR identified a focus on the importance of societal over individual solutions.^16^, ^17^, ^18^ No previous analyses of media reporting of sepsis have been identified.

This novel study aims to answer the following questions:

• What are the similarities and differences in how definitions and drivers of and solutions to AMR and sepsis are framed in UK newspapers?
• What do these media constructions of AMR and sepsis suggest for public understandings and for informing future public health communications on these issues?

## Methods

### Sampling

Six highly-circulated UK newspapers were selected, along with their Sunday editions^4^.^19^ The chosen publications comprised two broadsheet, two middle market and two tabloid newspapers, a typology used in other analyses of newspaper media discourse to represent a range of readership profiles diverse in age, social class and political ideology.^20^

### Searching

The Nexis database was searched to identify articles published on all available dates until 30.6.18 that included three or more mentions of any of the following terms: “sepsis”; “septicaemia”; “blood poisoning”; “AMR”; “antimicrobial resistance”; and “antibiotic resistance”. The following exclusion criteria were applied: regional editions; letters; obituaries, book, television and theatre reviews and articles whose main focus was not AMR or sepsis. Articles about sepsis were excluded if they were primarily about meningitis or the meningococcal vaccine, as these form a separate narrative around eligibility criteria and ethical debates about obtaining vaccines privately. Articles about sepsis/septicaemia that mentioned meningitis as an associated condition were included where the primary topic was sepsis.

### Developing the coding frame

A framework was constructed to enable systematic coding of the manifest content of articles; this refers to overt meanings easily identifiable within the text and readily quantifiable. Codes were devised using a combined approach of applying a framework of elements of reporting identified as having capacity to influence audience perception of risk, and themes that emerged through close reading of randomly-selected articles from each year until relevant novel themes ceased to emerge.^21^

LR, SH and LM^c^ discussed how to group emergent codes and categorised them according to three broad themes:

• How have the problems of sepsis and AMR been defined?
• What are the reported drivers of sepsis and AMR?
• What are the reported solutions to sepsis and AMR and how are they presented?

A further category was added to capture whether a case history was included, i.e. reference to an identifiable individual personally affected by either issue. A definitions sheet was produced and referred to throughout the coding process to ensure consistency.

### Assessing validity

After LR coded the first 200 articles, 20 were randomly selected and double-coded by CP. During the double-coding process, CP and LR discussed their interpretations of codes to identify areas of ambiguity. A Cohen's kappa test of interrater agreement was performed; this takes into account the level of agreement that might occur by chance, producing a test statistic between −1 and 1, with values in excess of 0.8 typically classified as indicating almost perfect agreement.^22^ Themes with a score lower than 0.8 were discarded or the coding definition revised for clarity. All articles were then re-coded by LR using the revised definitions.

### Quantitative analysis

The remaining articles were coded by LR and data entered into SPSS V.21. Linear regression was used to determine the statistical significance of trends in publication over time. Chi-square tests of association were performed to analyse differences in allocation of codes according to subject, with statistical significance set at *p* < 0.05. Examples of typical quotations are provided to illustrate findings.

## Findings

An overview of articles is presented (Table 1) followed by results of the comparative analysis of themes present in articles about AMR and sepsis (Table 2) and illustrative quotes (Table 3).

**Table 1:** Summary of articles in sample.

Title	Sepsis (*n* = 415)	%	AMR (*n* = 201)	%
Broadsheet				
Guardian/Observer	48	11.6	87	43.3
Telegraph/Sunday Telegraph	50	12.0	51	25.4
Middle market				
Daily Mail/Mail on Sunday	202	48.7	42	20.9
Express/Sunday Express	31	7.5	15	7.5
Tabloid				
Mirror/Sunday Mirror	37	8.9	3	1.5
Sun/News of the World	47	11.3	3	1.5

**Table 2:** Comparison of themes within articles about sepsis/AMR.

	Sepsis (*n* = 415)		AMR (*n* = 201)		p-value of chi square test of association between themes for sepsis/AMR
	n	%	n	%	
Problem definition					
Article about sepsis references AMR or vice versa	29	7.0	27	13.4	0·009
States rates within UK	173	41.7	48	23.9	<0.001
States rates out with UK	14	3.4	52	25.9	<0·001
States greatest health risk is in future	6	1.4	88	43.8	<0·001
States associated economic impact	20	4.8	22	10.9	0·005
Compares issue to other health conditions	55	13.3	22	10.9	0·417
Presents the following as ‘at risk’ group:					
Infants or children	50	12.0	6	3·0	<0·001
Pregnant women	8	1·9	0	0	0·048
Elderly	34	8.2	4	2.0	0·003
Individuals with pre-existing health conditions	44	10.6	22	10.9	0·897
Everyone	23	5.5	3	1.5	0·019
Problem drivers					
Human healthcare – systemic factors	211	50.8	99	49.3	0·711
Human healthcare – behaviour of identifiable individual health professional	167	40.2	1	0.5	<0·001
Behaviour of the public	6	1.4	39	19.4	<0·001
Actions of farming or food industry	6	1.4	83	41.3	<0·001
Actions of pharmaceutical industry	11	2.7	60	29.9	<0·001
Actions of Government	24	5.8	18	9.0	0·143
Solutions					
Awareness-raising	153	36.9	33	16.4	<0·001
Technical (e.g. diagnostic tests or drug development)	52	12.5	103	51.2	<0·001
Systemic (e.g. changes to protocols or regulation)	104	25.1	123	61.2	<0·001
Reduction in unnecessary prescribing of antibiotics in humans	10	2.4	86	42.8	<0·001
Treatment with early antibiotics	86	20.7	2	1.0	<0·001
Contains case history					
	311	74.9	14	7.0	<0.001

**Table 3:** Typical quotations illustrating UK newspapers’ framing of sepsis and AMR.

	Sepsis	AMR
Problem definition	‘Sepsis, a stealthy killer that claims 44, 000 lives a year in the UK, is the big dirty secret at the heart of the NHS.’ (Daily Mail, 9 February, 2016).	‘Failure to tackle drug-resistant infections will lead to at least 10 million extra deaths a year… by 2050… the world's most populous countries, India and China, face 2 million… Africa as a continent will suffer greatly.’ (The Guardian, 11 December, 2014).
Problem drivers	‘A three-year old boy… died from sepsis poisoning after medical staff failed to assess him properly… when [he] finally reached hospital there was a three-hour delay before he was given the antibiotics that could have saved his life.’ (The Guardian, 26 June, 2014).	‘Doctors must be banned from prescribing antibiotics without test results proving they are needed, the country's superbugs tsar demands… he accused doctors of doling out antibiotics ‘like sweets’ and called for severe curbs to control their use.’ (Daily Mail, 19 May, 2016).
Solutions	“It is vital the message gets out to people… [it] should centre on simply getting the word sepsis into everyone's minds… they should ask their doctor: “Could this be sepsis?” … if lives are to be saved, it is crucial to act quickly, within the “golden hour” of the condition setting in”(Daily Mail, 14 June, 2016).	‘Only global co-operation can fight antibiotic resistance… the war [on AMR] will require real action from governments around the world to build international alliances, engage with behavioural science and rethink their relationship with the pharmaceutical industry.’ (The Observer, 6 July, 2014).

## Search results

**Fig. 1:**
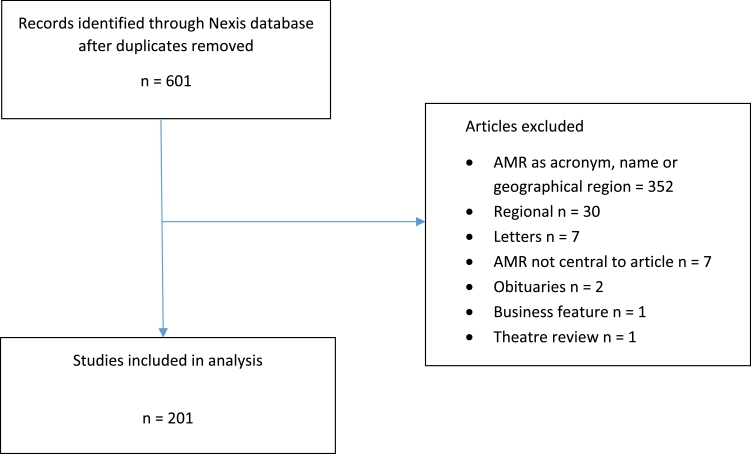
Search process (AMR).

**Fig. 2:**
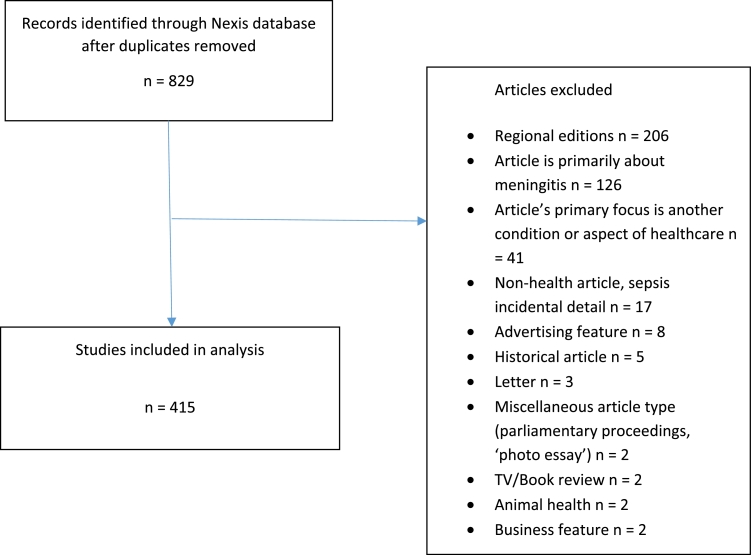
Search process (sepsis).

Figs. 1 and 2 show the results of the search processes with the number of exclusions at each stage. The number of articles eligible for analysis was 616. Categories were mutually exclusive i.e. no articles were included in both samples.

## Location of articles and time trends in publication

Articles about AMR were more frequent in broadsheet publications, while for sepsis middle market publications dominated (Table 1).

**Fig. 3:**
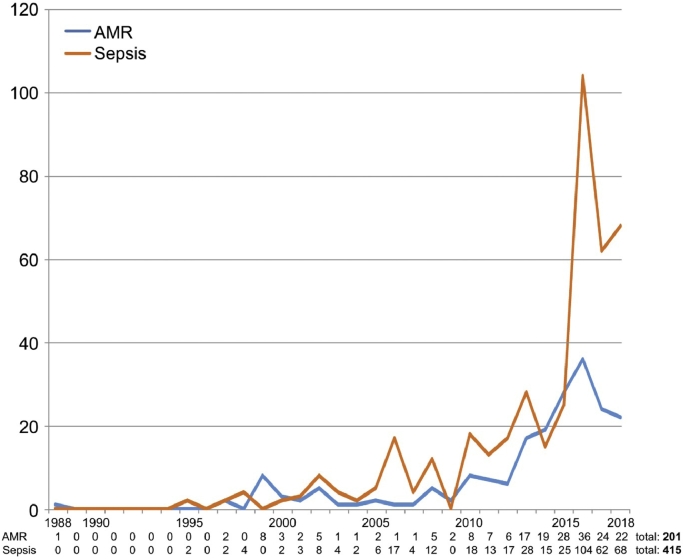
Trends in articles published by year and subject.

The frequency of articles increased over time (Fig. 3), with the earliest article identified published on 30th January 1988. A simple linear regression demonstrated that progressive publication quarter was a statistically significant predictor of number of articles published for both sepsis (coefficient 0·614, *p* < 0·001) and AMR (coefficient 0·620, *p* < 0·001). Key events in relation to reporting of AMR were the release of publications by the Review on AMR, beginning in December 2014, and first reports of resistance to colistin (referred to as ‘antibiotics of last resort’) in food animals in November 2015.^23^ For sepsis, an increase in reporting followed publication of the findings of the 2016 enquiry into the death of William Mead. Following this, and backed by the Sepsis Trust and families of individuals affected by sepsis, including William's parents, the Daily Mail launched a campaign entitled ‘End the Sepsis Scandal’, publishing a host of articles that detailed similar cases, both current and retrospective. Reporting was sustained in the early part of 2018, in part associated with publicity surrounding the General Medical Council's (GMC) ruling to remove a junior doctor (Dr Bawa Garba) from the medical register after she was convicted of manslaughter by gross negligence, having failed to diagnose sepsis in a six-year old boy.^24^ This decision provoked anger within the medical community, concerned about its implications for promoting an organisational culture that encourages transparency and learning, and has subsequently been overturned by the court of appeal, a decision welcomed by professional organisations including the British Medical Association (BMA).^25^

## Framing sepsis and AMR

Table 2 illustrates the frequency of each thematic code within articles about sepsis and AMR, and indicates the *p*-value of a chi-square test of association between each code and article topic. Table 3 provides examples of typical quotes that illustrate how sepsis and AMR are defined and how their drivers and solutions are framed.

## Problem definition

Sepsis and AMR were largely presented as separate issues, with only a minority (9%) of the 616 articles included in the analysis mentioning both. Of the 201 articles that were primarily about AMR, only 27 (13.4%) mentioned sepsis, septicaemia or blood poisoning. For sepsis, the crossover was even lower, with only 29 (7%) of the 415 articles referencing AMR, antimicrobial resistance or antibiotic resistance. Sepsis was most often presented in relation to its incidence within the UK rather than globally (41.7% vs. 3.4%) whereas AMR was described in both a UK (23.9%) and global (25.9%) context and frequently presented predominantly as a future risk to population health (43.8%) (Table 3). Articles about sepsis were four times as likely to present infants and children and the elderly as groups within the population who are at increased risk, but also more often presented sepsis as a condition that is indiscriminate and can affect all individuals in a population (5.5% vs. 1.5%), regardless of pre-existing risk factors.

## Problem drivers

Similar proportions of articles presented systemic healthcare factors as drivers of sepsis (50.8%) and AMR (49.3%), referencing failures in diagnosis and treatment delays and overprescribing of antibiotics respectively (Table 3). Over one-third (40.2%) of articles about sepsis referred to the actions of potentially identifiable healthcare staff as at fault. In contrast, only one article about AMR referenced actions of an individual health professional and this was in relation to negative impacts resulting from pressure not to prescribe antibiotics. As anticipated, articles about AMR implicated the actions of the farming, food and pharmaceutical industries, highlighting the complexity of its causes. Articles about AMR more frequently identified the behaviour of the public as a problem driver (19.4% vs. 1.4%), referencing patients’ ability to influence prescribing and the contribution of global travel and health tourism to the spread of drug resistance.

## Solutions

Solutions to AMR were presentedas technical (51.2%), i.e. the development of new drugs or diagnostic tests or systemic (61.2%), e.g. changes in how research and development into new antimicrobials is funded, with the need for international collaboration highlighted, which is in keeping with the drivers presented (Table 3). For sepsis, systemic and technical solutions were also proposed, e.g. improved management pathways and rapid diagnostic tests, but the need for increased awareness among the pubic and health professionals was identified more frequently than for AMR (36.9% v. 16.4%) and presented as key to improving outcomes. Just as few articles referenced both AMR and sepsis, so the potential conflict between solutions to each was overlooked, with only 2.4% of articles about sepsis identifying the need to reduce unnecessary prescribing.

## Case histories

The majority of articles about sepsis (74.9%) included narratives about named individuals who had been affected, many describing death or serious disability resulting from misdiagnoses by doctors or other healthcare staff. Within these 311 articles, 45.7% referred to a baby or child and, of note, 23.5% involved high-profile individuals from diverse fields including sport, music, acting and politics. In contrast, only 14 articles about AMR (7.0%) identified affected individuals, tending to use their stories in brief and for illustrative purposes rather than as a primary focus. Only four individuals who had died as a result of resistant infections were identified, two of whom were from outside the UK.^26^^,^^27^

## Discussion

Our study identifies for the first time potentially conflicting health messages about antibiotic use through differential framing of AMR and sepsis within UK newspapers. AMR was presented as a global issue, driven by multiple sectors, whose main health impact will be in the future. The need for co-ordinated actions between policymakers and healthcare, farming and pharmaceutical industries was highlighted, consistent with previous analyses of newspaper reporting of AMR that identified a focus on societal over individual solutions.^16^^–^^18^ In contrast, sepsis was most often presented in the context of its current impact within UK healthcare, driven by both systemic and individual failings in diagnosis and management. Proposed solutions focused on empowering the public through greater awareness, emphasising the difference that early antibiotic treatment has on outcomes. Few articles balanced this message with the importance of rational prescribing, with only a minority of articles about sepsis referring to AMR and vice versa.

The majority of articles about sepsis identified individuals that had been personally affected, often children or celebrities, frequently identifying where action or inaction of health professionals contributed to negative outcomes. These are all features of reporting that have been demonstrated to have the capacity to increase public interest in media discourse about health risks.^21^ Such personal narratives were rarely present in articles about AMR, which instead focused on projections about its potential impact at population level. Qualitative research on public perceptions of AMR has identified that current ways of communicating fail to demonstrate impact at a personal level. Our findings provide quantitative evidence of this, with only a small minority of articles documenting the impact of resistant infections on individuals.

Given the influence of media representations on public understandings of health issues, media reporting of sepsis may impact perceptions of the risks associated with non-treatment with antibiotics.^15^^,^^28^ Sepsis is a heterogeneous condition and the narratives that characterise media reports may not be typical of the estimated 44,000 deaths that occur in the UK each year, many of which occur in older adults who have complex co-morbidities.^5^ Frequency of print media reporting has been shown to influence lay estimation of mortality risk, leading to a tendency to overestimate the likelihood of death from rare or dramatic causes.^29^ If emotive media constructions of sepsis have the capacity to alter perception of risks associated with common illnesses they may impact on expectations about antibiotics, which is known to influence prescribing, particularly for children.^30^ For health professionals, AMR is a low priority compared to the potential harm caused by withholding antibiotics, and no existing evidence demonstrates that antimicrobial prescribing in primary care can be safely reduced without impacting on incidence of sepsis.^12^^,^^31^

Fear of having ‘missed’ a diagnosis has been identified as a key pressure in primary care, alongside implementing new clinical guidelines and the impact of public health campaigns.^32^ The consequences of greater sepsis awareness must be considered in the context of a healthcare system experiencing increasing demand without corresponding increases in capacity, including a disproportionate rise in consultations by the very young and elderly.^6^ In primary care, where over 80% of antibiotics are prescribed, there remains a lack of useful rapid diagnostic tests, and prescribing decisions are frequently influenced by factors other than clinical signs, including doctor-patient relationships, previous negative experiences of not prescribing and ‘gut instinct’ about whether a patient is unwell.^33^, ^34^, ^35^ The UK Review on AMR identified the need for a global awareness campaign to reduce public demand for antibiotics and unnecessary prescribing, but it is unrealistic to make these calls without acknowledging the impact of sustained exposure to alarming accounts in the media about the consequences of treatment delays.^2^ It is possible that recent high-profile reporting of the GMC's now overturned ruling against Dr Bawa Garba, centring on her failure to diagnose sepsis, may contribute to a culture of defensive practice.

The public health implications of our findings are twofold. Firstly, health communications about sepsis in the media must balance messages about the importance of early treatment with antibiotics with information about risks of overuse, particularly of broad-spectrum antibiotics in the absence of microbiological confirmation of sensitivities. The impact of sepsis awareness campaigns on health outcomes must be carefully evaluated and should include consideration of the potential for unintended effects, not only on unnecessary prescribing, but also on patient and prescriber anxiety. At present, the public are exposed to conflicting messages about antibiotic use, on the one hand being asked to stop demanding antibiotics to manage viral symptoms and on the other being encouraged to challenge health professionals about whether any symptoms consistent with infection could indicate sepsis. Increased awareness is essential to ensure that treatment is rapidly established when clinically indicated but, for the majority of patients who present to primary care, decisions about antibiotic prescribing are subject to uncertainty.^9^ Greater clarity is required about how prescribers can adhere to antimicrobial stewardship practices without leaving themselves vulnerable to legal challenge. Uniting media discourses about sepsis and AMR would better equip clinicians to initiate dialogue with patients about the rationale behind prescribing decisions in a context that provides transparency about the potential for clinical deterioration and with clear advice about when they should re-attend.

Secondly, learning from sepsis awareness initiatives may help inform communications about the population health risks associated with AMR. The Sepsis Trust has worked closely with the UK media, bringing stories about individuals affected by sepsis into the public domain in a way that is immediately accessible, even providing advice on how television writers can use storylines about sepsis to improve awareness.^36^ At present, there is a lack of similar media presence highlighting the human impact of drug resistant infections, with communications focusing on potential future global and economic health impacts, despite an estimated 700,000 individuals who currently die globally as a result of resistant infections each year. Efforts to secure AMR as an issue that has relevance for the public has been hampered by a perceived lack of meaningful actions that can be taken at an individual level, with reducing demand for antibiotics just one facet of a complex strategy that requires internationally co-ordinated responses by policymakers.^1^ Securing public support is essential to drive the political will necessary to deliver the individual components of this strategy, including the development of novel therapies and rapid diagnostic tests that will enable safe reduction in unnecessary prescribing. The relative lack of funding from the charity sector for research into AMR in comparison to higher profile conditions has been highlighted from within the pharmaceutical industry, with calls for the public to realise the importance of antibiotic research.^37^ It may be possible to alter public perceptions of AMR by highlighting the human cost of drug resistance via a similar strategy to that used by campaigners for sepsis awareness.

Our study benefited from no date restrictions, limiting potential bias from short-term variation in reporting in response to key events. Double coding a subsample of articles provided assurance of internal validity. While the sample did not include online news sources, it has been demonstrated that these are broadly similar in content to print news.^38^ As is inherent to media content analysis, our research design can identify media representations of the issue, but not determine audiences’ responses to those messages; while we can hypothesise about the potential impact of media content on attitudes to antibiotics, demonstrating whether this is the case will require further research involving the public and health professionals. Similarly, demonstrating any impact on antibiotic use requires analysis of prescribing data alongside consideration of other influences, for example increased prevalence of bacterial infections in the community.

We have demonstrated for the first time that AMR and sepsis are largely constructed as separate issues in UK newspapers, with a resulting lack of clarity about optimum antibiotic use. This disjunction compromises messages about the importance of balancing the need for sufficient access to antibiotics to protect individual health outcomes with safeguarding population health in the longer term. We suggest that media narratives about drug resistant infections must be brought in line with those about sepsis in order to raise public consciousness of AMR as an issue that directly impacts on individual health outcomes.

## Declarations of interest

None.

## Footnotes

1 The Review on AMR was an expert panel commissioned by the UK Government in 2014 tasked with analysing the economic and social impacts of AMR and proposing solutions to these.

2 NICE is a UK non-departmental public body that sponsored by but separate from the Department of Health that produces evidence-based guidance for health practitioners.

3 NHS England is a non-departmental public body sponsored by the Department of Health that oversees planning and delivery of health services in England.

4 Note that circulation of the News of the World ceased in July 2011; the Sun on Sunday was launched by the same newsgroup in 2012, but is unavailable on the Nexis database.
